# Predictive tissue markers in testicular germ cell tumors: Immunohistochemical expression of MLH1 and REV-7 proteins

**DOI:** 10.1007/s11255-023-03933-2

**Published:** 2024-01-27

**Authors:** Theodoros Spinos, Eleni Zografos, Konstantinos Koutsoukos, Flora Zagouri, Christos Kosmas, Tatiana S. Driva, Dimitrios Goutas, Charikleia Gakiopoulou, George Agrogiannis, Eirini Theochari, Chara Tzavara, Andreas C. Lazaris

**Affiliations:** 1 First Department of Pathology, School of Medicine, National and Kapodistrian University of Athens, 75 Mikras Asias Str., 115 27, Goudi, Athens, Greece; 2 Oncology Unit, Department of Clinical Therapeutics, National and Apodistrian University of Athens, Alexandra Hospital, Athens, Greece; 3 Department of Medical Oncology, Hematopoietic Cell Transplant Unit, Metaxa Memorial Cancer Hospital, Piraeus, Greece; 4 Department of Biostatistics, Medical School, National and Kapodistrian University of Athens, Athens, Greece

**Keywords:** Testicular germ cell tumors, Cisplatin, Chemoresistance, MLH1, REV-7, Predictive tissue markers

## Abstract

**Purpose:**

Testicular Germ Cell Tumors (TGCTs) are the most frequent solid malignancies in young adult men. Regardless of differences in their cell of origin, all TGCTs are considered highly curable malignancies. However, approximately 3–5% of all TGCTs do not respond to platinum-based chemotherapies. The purpose of our paper is to investigate whether immunohistochemical expression of MLH1 and REV-7 can be used as predictive tissue markers for TGCTs.

**Material and Methods:**

The main demographic and clinicopathological characteristics of 64 male patients with TGCTs who underwent orchiectomy from 2007 to 2022 were retrospectively obtained from two large Oncology Clinics in Greece. Both patients with chemosensitive and chemoresistant disease were included. Immunohistochemical staining for MLH1 and REV-7 proteins was applied in specimens of these patients.

**Results:**

31 seminomas and 33 non-seminomas were included. 48 patients had chemosensitive disease, while 16 had chemoresistant disease. 53 specimens showed preserved MLH1 expression, while 11 specimens had lost MLH1 expression. Expression of MLH1 was only significantly associated with patients’ age. 16 specimens showed positive REV-7 expression, while 48 specimens were REV-7 negative. Interestingly, 50% of patients with chemoresistant disease and 16,7% of patients with chemosensitive disease were REV-7 positive. This difference was statistically significant. Moreover, REV-7 positivity was significantly associated with chemoresistance, various clinicopathological parameters and patients’ prognosis and survival.

**Conclusion:**

Loss of MLH1 expression was only found to be significantly associated with lower patients’ age. Positive immunohistochemical REV-7 expression was significantly associated with various clinicopathological parameters, while it was also associated with significantly lower survival and greater hazard. REV-7 positive percentages were significantly higher in patients with chemoresistant disease. Our findings imply that immunohistochemical staining for REV-7 could potentially be used as a predictive tissue marker for TGCT tumors. Moreover, targeting of REV-7 protein, could represent a potential therapeutic strategy for chemoresistant TGCT cases. The implementation of well-designed studies on a larger scale is of utmost importance, in order to draw safer conclusions. Additional studies are needed so as to draw safer conclusions.

**Supplementary Information:**

The online version contains supplementary material available at 10.1007/s11255-023-03933-2.

## Introduction

Although testicular cancer is rare in the general population, it represents the most frequent solid malignant tumor in young adult men [1]. Testicular Germ Cell Tumors (TGCTs) account for most cases of testicular cancer diagnoses [2]. TGCTs can be further divided into seminomas, which are more frequent in the fourth decade of life and non-seminomas, which are more frequent in the third decade of life [3, 4]. The gold standard treatment of TGCTs is radical orchiectomy, which in most cases is combined with chemotherapy, radiotherapy or retroperitoneal lymph node dissection (RPLND) [2]. Regardless of differences in their cell of origin, all TGCTs are considered highly curable malignancies. Indeed, although many cases of TGCTs are metastatic at diagnosis, their survival rates are far better than that of most other malignancies and, in some cases, even exceed 90% [5]. Chemotherapy regimens with cisplatin and etoposide, along with or without bleomycin, have been associated with maximal total remission and cure rates [6]. Nevertheless, approximately 3–5% of all TGCTs and 10–15% of initially advanced TGCTs do not respond to platinum-based chemotherapies, often resulting in patients’ death. The etiology of treatment failure is considered the development of chemotherapy resistance [7]. For all these reasons, the treatment of chemoresistant TGCTs represents a challenge for both clinicians and researchers, while the development of predictive tissue markers is of vital importance.

The DNA Mismatch repair (MMR) system, consisting of several proteins, recognizes and repairs incorrect DNA structures into the cell nucleus, originating from errors during DNA replication and recombination. When the MMR system functions irregularly, mutation rates increase, as evidenced by alterations in repetitive DNA sequences, also called microsatellite instability (MSI) [8]. The MLH1 protein is an important part of the MMR system in human cells [9]. Another intracellular mechanism which has been developed in an effort to manage DNA damage, caused my various endogenous and exogenous factors, including chemotherapy drugs such as cisplatin, is called Translesion DNA synthesis (TLS) [10]. DNA Polymerase ζ (Polζ) is one of its most important ingredients and skips DNA-damaged lesions [11–13]. The REV-7 protein (also known as MAD2L2 and MAD2B) is a subunit of Polζ [14]. Apart from its significance in TLS and cell cycle checkpoint, REV-7 has also been involved in several other important biological procedures, such as the Homologous Recombination (HR) and the DNA Double-Strand Break (DSB) repair [15, 16]. Hypothesizing that these mechanisms could be engaged in the development of chemoresistance of TGCTs, we reported the immunohistochemical expression of MLH1 and REV-7 proteins in TGCT tissues from patients with both chemosensitive and chemoresistant disease. Moreover, we studied the association of their expression with various clinicopathological parameters and patients’ prognosis and overall survival. The purpose of our paper is to investigate whether MLH1 and REV-7 can be used as predictive tissue markers for TGCT tumors.

## Material and methods

### Patients and clinical samples

Data concerning 64 male patients who have been diagnosed with TGCTs were retrospectively obtained from two national leading Cancer Centers in Greece. Patients had undergone orchiectomy from 2007 to 2022, in five Hospitals in Greece. Orchiectomy specimens were obtained from the Pathology Laboratories of these Hospitals. Both patients with chemosensitive and chemoresistant disease were included. Patients with chemosensitive disease were considered those who either presented with metastatic or advanced disease at diagnosis and showed complete response after chemotherapy, or presented with local disease and were offered chemotherapy, showing no local recurrence or metastases during follow-up. Moreover, patients with chemosensitive disease were considered those who followed a surveillance program after local disease diagnosis and underwent chemotherapy after relapse or metastasis during follow-up, showing complete response. On the contrary, patients with chemoresistant disease were considered those with advanced disease that had viable residual disease following chemotherapy, or did not respond to chemotherapy. Finally, patients with chemoresistant disease were also considered those who relapsed after initial response to chemotherapy. The study was approved by the Ethical Committees of the participating hospitals.

The pathology reports of all 64 orchiectomies were collected. Based on their protocol numbers, the hematoxylin and eosin (H&E) stained slides from all sections of these cases were reviewed and the most appropriate section was selected. A representative slide was selected for each case. In the case of mixed non-seminomatous tumors, emphasis was given into choosing a section that included all the components of the tumor. In uncertain cases, a second slide was also selected. The paraffin blocks of all selected slides were then retrieved from the Archives of the Laboratories. In total, 68 paraffin blocks were collected. From each selected paraffin block, four new sections were cut and tissues were placed on special slides, which were suitable for immunohistochemical analysis. In each case, one section was processed with hematoxylin and eosin (H&E), while the remaining three were unstained sections for immunohistochemical analysis (one for MLH1 processing, one for REV-7 processing and one was stored as a back-up).

### Immunohistochemistry

Immunohistochemical detection of the examined proteins was performed on 4-μm-thick formalin-fixed paraffin sections which underwent overnight heating at 37 ^ο^C. Deparaffinization, rehydration and antigen retrieval were performed using an automated module (PT Link, Dako) for 20 min at 96 °C, with the reagent EnVision FLEX Target Retrieval Solution High pH (50x) (DAKO Envision FLEX kit, DAKO, Carpinteria, CA) for MLH1 and EnVision FLEX Target Retrieval Solution Low pH (50x) (DAKO Envision FLEX kit, DAKO, Carpinteria, CA) for REV-7. To block endogenous peroxidase activity 0.3%, hydrogen peroxide was applied for 15 min in dark environment. Sections were rinsed with Tris Buffered Saline (TBS) and Tissue Primer was applied for 5 min. After rinsing with TBS, the sections were incubated with Background Blocker for 5 min. These steps were performed according to manufacturer protocol [Mouse/Rabbit PolyVue Plus HRP/DAB Detection System (Diagnostic BioSystems, Pleasanton, CA)]. This step was followed by overnight incubation of the sections at 4 °C with the primary antibodies: anti-MLH-1 (GeneAb, GenomeMe, Richmond, Canada) at a dilution of 1:100 and anti-Rev7 (Abcam, Cambridge, UK) at a dilution of 1:300. For visualization, a two-step technique [(Mouse/Rabbit PolyVue Plus HRP/DAB Detection System (Diagnostic BioSystems, Pleasanton, CA)] was performed, according to manufacturer protocol, with diaminobenizidine as a chromogen. Hematoxylin was used to counterstain the sections. Two independent reviewers evaluated the immunohistochemical staining in selected slides. REV-7 immunopositivity was interpreted as any positive cytoplasmic and/or nuclear staining in more than 51% of the neoplastic cells. (Cut off point was set arbitrarily). MLH1 was interpreted as positive (preserved expression) based on the preserved expression in 100% of neoplastic cells or negative (loss of expression) based on the complete MLH1 loss in the entire tumor area.

### Statistical analysis

Variables were first tested for normality using the Kolmogorov–Smirnov criterion. Quantitative variables were expressed as mean (Standard Deviation) or as median (interquantile range). Categorical variables were expressed as absolute and relative frequencies. For the comparison of proportions chi-square and Fisher’s exact tests were used. Independent samples Student’s t-tests were used for the comparison of quantitative variables between two groups when the distribution was normal and Mann–Whitney tests were used for the comparison of quantitative variables between two groups when the distribution was not normal. Multivariate Cox proportional-hazard model in a stepwise method (p for entry 0.05, p for removal 0.10) was used in order to determine the independent predictors for survival. The assumption of proportional hazards was evaluated by testing for interaction with a continuous time variable. Hazard ratios (HR) with 95% confidence intervals (95% CI) were computed from the Cox regression analyses. Kaplan – Meier survival estimates were graphed over the follow-up period. All reported p values are two-tailed. Statistical significance was set at p < 0.05 and analyses were conducted using SPSS statistical software (version 26.0).

## Results

Our patient cohort consisted of 64 male patients, with a mean age of 36.2 years (SD = 11.3 years), who had undergone orchiectomy and were diagnosed with testicular germ cell tumors (TGCTs). Patients’ demographics and clinical characteristics are presented in Table 1. 16 patients (25%) had chemoresistant disease, while 48 patients (75%) had chemosensitive disease, according to the abovementioned criteria. 31 patients (48.4%) were diagnosed with pure seminoma, 25 (39.1%) with pure non-seminoma and 8 (12.5%) with mixed non-seminomatous tumors (consisting of both seminoma and non-seminoma characteristics). Of those, 25 (39.1%) patients had high-risk factors for stage I seminoma (tumor size > 4 cm or rete testis infiltration), 25 (39.1%) had high-risk factors for stage I non-seminoma (lymphovascular invasion, LVI) and 14 (21.9%) had no high-risk factors at all. Orchiectomy was performed on the right side for 31 (48.4%) patients, on the left for 28 (43.8%) and bilateral for five (7.8%) patients. Furthermore, half of the patients (50%) had T2 disease, 75.5% had N0 disease and 54.2% had M0 disease at presentation, according to the TNM (tumor, nodes, metastasis) classification system. The majority of patients (48.4%) had been diagnosed with stage I disease, according to the International Germ Cell Cancer Collaborative Group (IGCCCG). 35 patients (54.7%) had metastases (stage II or III, according to TNM staging) at diagnosis. The most common site of metastasis was the retroperitoneal lymph nodes (RPLNs, n = 30). Other sites were mediastinal lymph nodes (n = 1), inguinal lymph nodes (n = 1), lungs (n = 12), liver (n = 1) and adrenal glands (n = 2). Median Lactate Dehydrogenase (LDH), Alpha-fetoprotein (AFP) and Beta Subunit Human Chorionic (βHCG) values at diagnosis were 268.5 (190.5 ─ 447) IU/L, 3.4 (1.8 ─ 56.4) ng/ml and 1.8 (1 ─ 206.1) mIU/mL, respectively. 41.7%, 30% and 32% of patients had abnormal LDH, AFP and βHCG values at diagnosis, respectively.

**Table 1:** Patients demographics and clinical characteristics

	*n* (%)
Chemoresistant disease	16 (25.0)
Histological type	
Pure non-seminoma	25 (39.1)
Pure seminoma	31 (48.4)
Mixed non-seminomatous tumor	8 (12.5)
Seminoma (%), mean (SD)	54.08 (47.05)
Choriocarcinoma (%), mean (SD)	0.78 (3.24)
Yolk sac tumor (%), mean (SD)	7.48 (20.16)
Teratoma (%), mean (SD)	13.13 (27.49)
Embryonal carcinoma (%), mean (SD)	24.69 (35.82)
High-risk factors	
No	14 (21.9)
Yes (for stage I seminoma)	25 (39.1)
Yes (for stage I non-seminoma)	25 (39.1)
Side of Orchiectomy	
Right	31 (48.4)
Left	28 (43.8)
Both	5 (7.8)
T (Tumor)	
1	17 (26.6)
2	32 (50.0)
3	15 (23.4)
N (Nodes)	
0	40 (75.5)
1	4 (7.5)
2	5 (9.4)
3	4 (7.5)
M (Metastasis)	
0	32 (54.2)
1a	24 (40.7)
1b	3 (5.1)
1c	0 (0.0)
IGCCCG stage at diagnosis	
Ι	31 (48.4)
ΙΙ	10 (15.6)
ΙΙΙ	23 (35.9)
Metastasis at diagnosis	35 (54.7)
MLH1	
Preserved	53 (82.8)
Loss of expression	11 (17.2)
REV-7	
Negative	48 (75.0)
Positive	16 (25.0)

Table 2 provides information about patients’ treatment characteristics. Almost all patients (96.9%) had undergone chemotherapy after diagnosis, while none of them had undergone radiotherapy treatment. The most common chemotherapy regimen was BEP (Bleomycin, Etoposide and Platinum) being offered in 37 (56.9%) patients, followed by CARBO (Carboplatin) 7 AUC (Area Under the Curve) and CARBO 6 AUC. For 32 out of 62 (51.6%) patients who were offered chemotherapy, the regimen was characterized as advanced disease chemotherapy (51.6%), while for 30 (48.4%) it was characterized as adjuvant treatment. Out of 62 patients who received chemotherapy, 18 (29.0%) showed complete response, 3 (4.8%) partial response, 9 (14.5%) disease progression and 2 (3.2%) stable disease. After treatment, 17 (26.6%) patients had a residual disease > 1cm. The most common site of residual disease was the RPLNDs. Moreover, 9 (14.1%) patients presented with relapse during follow-up. Once again, the most common site of relapse was the RPLNDs.

**Table 2:** Treatment characteristics of included patients

	*n* (%)
Chemotherapy	62 (96.9)
Type of chemotherapy^1^	
Advanced disease	32 (51.6)
Adjuvant	30 (48.4)
Response to chemotherapy^1^	
Adjuvant	30 (48.4)
Complete response	18 (29.0)
Partial response	3 (4.8)
Disease progression	9 (14.5)
Stable disease	2 (3.2)
Radiotherapy	0 (0.0)
Residual Disease > 1 cm	17 (26.6)
RPLND or metasectomy^2^	13 (92.9)

^1^Refers to patients who had undergone chemotherapy

^2^Refers to patients with residual disease > 1cm

*RPLND* Retroperitoneal Lymph Node Dissection

**Fig. 1:**
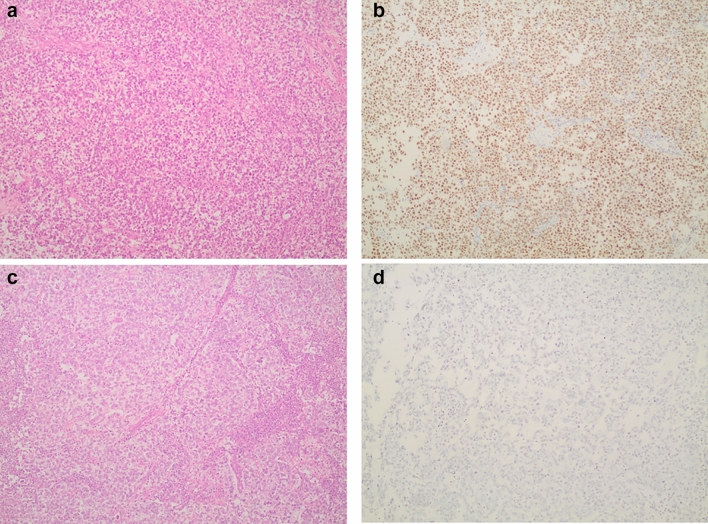
**a** Malignant cells of a pure seminoma (H&E,  × 200). **b** Evident MLH1 nuclear immunopositivity in seminomatous cells of the above tumor (× 200). **c** An embryonal carcinoma component of a mixed germ cell tumor (H&E, × 200). **d** Absence of MLH1 immunostaining in malignant cells of the above tumor (× 200)

Regarding MLH1 immunohistochemical staining of TGCT orchiectomy tissues, 53 specimens (82.8%) showed preserved MLH1 expression in nuclei of cancer cells, while 11 (17.2%) specimens had lost MLH1 expression. 25% of patients with chemoresistant disease did not express MLH1 in their pathology specimens, while only 14.6% of chemosensitive patients had loss of MLH1 expression. However, this difference was not statistically significant. Figure 1 shows representative images of MLH1 immunohistochemical staining in TGCTs from our sample. MLH1 expression was studied in correlation to patients’ baseline characteristics, prognosis and survival and to various clinicopathological factors. It was only found to be significantly associated with patients’ age. More specifically, in cases where MLH1 immunohistochemical expression was preserved, the mean age was 38.4 years-old (SD = 10.6 years-old), while in cases where MLH1 expression was lost, the mean age was significantly lower and equal to 25.6 years-old (SD = 8.3 years), p < 0.001. Moreover, MLH1 was not significantly associated with patients’ relapse (p > 0.999), probability of being disease-free at last follow-up (p = 0.683) and survival (p = 0.137).

**Fig. 2:**
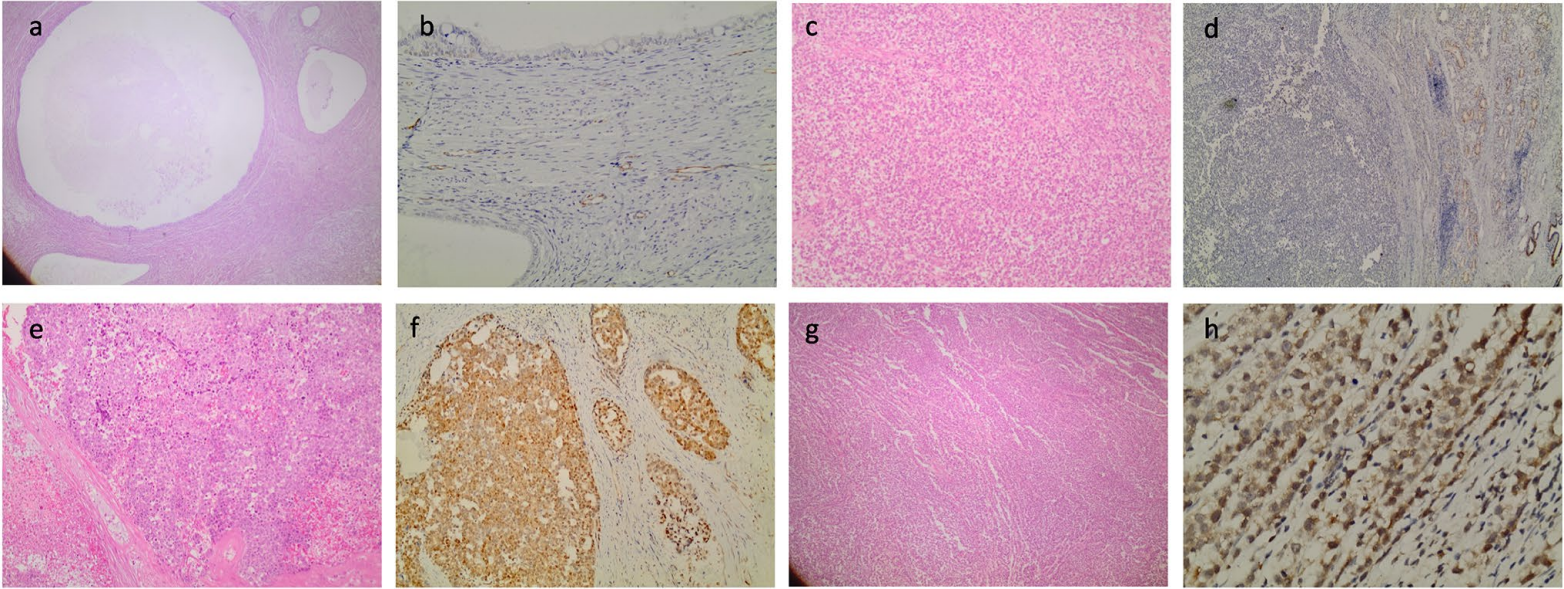
**a** Teratomatous elements of a non-seminoma tumor, containing 50% yolk sac tumor, 20% choriocarcinoma and 30% teratoma (H&E, × 200). **b** Negative REV-7 immunostaining of the above tumor with some positivity in entrapped tubules (X200). **c** Malignant cells of a pure seminoma (H&E, × 200). **d** Negative REV-7 immunostaining in the above seminoma with some positivity in adjacent tubular structures (× 200). **e** Mixed germ cell tumor comprising of 95% embryonal cell carcinoma and 5% yolk sac tumor (H&E, × 200). **f** Positive REV-7 immunostaining in malignant cells of the above tumor (× 200). **g** Another tumor of pure seminoma type (H&E, × 200). **h** Evident REV-7 immunopositivity in seminomatous cells’ nuclei and cytoplasm (× 400).

**Fig. 3:**
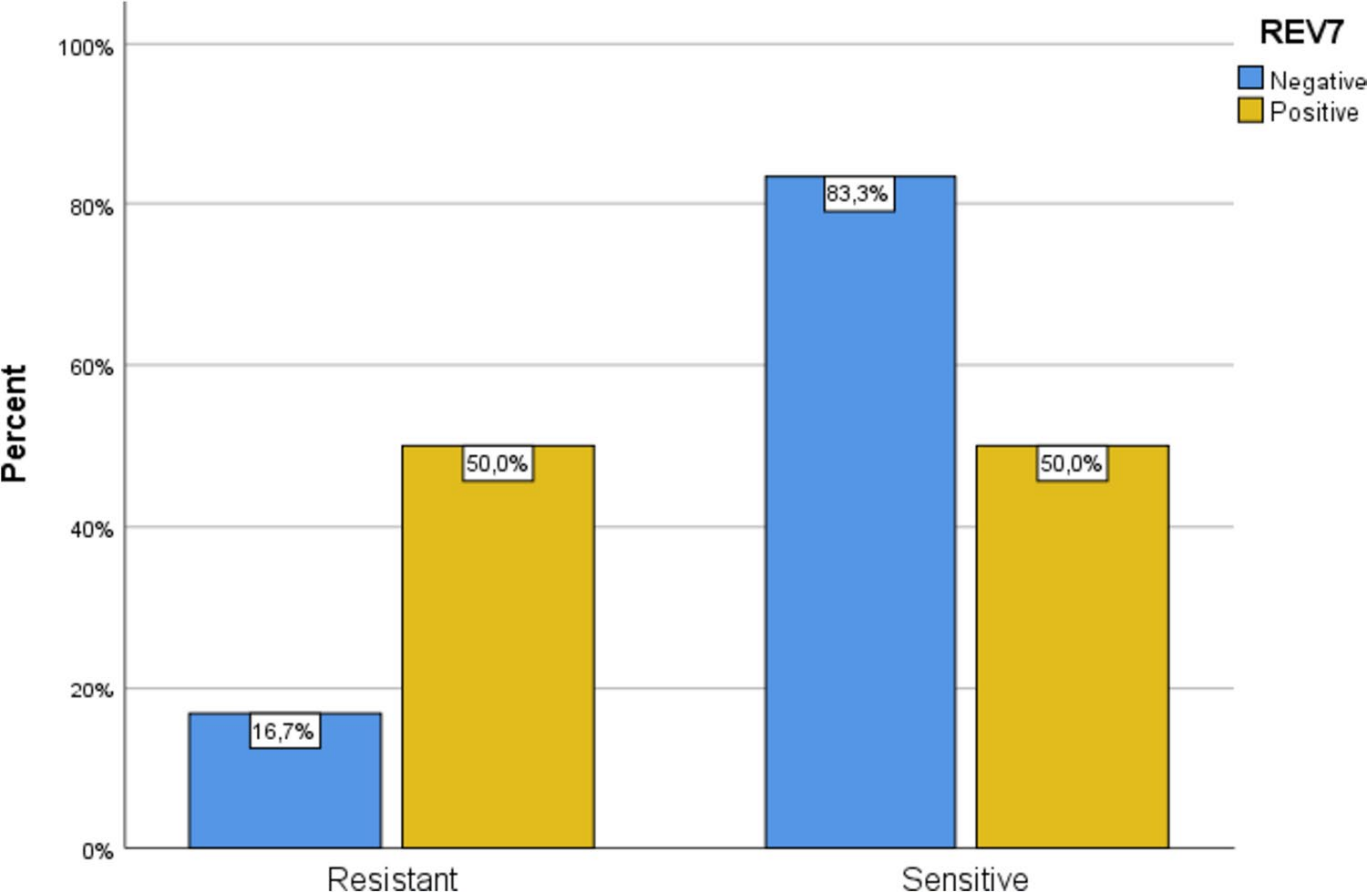
Percentages of REV-7 positive and negative specimens in patients with chemoresistant and chemosensitive disease

The association of REV-7 immunohistochemical expression with participants’ demographics and clinical characteristics is presented in Table 3. Figure 2 shows representative images of REV-7 immunohistochemical staining in TGCTs of our sample. REV-7 positive patients were considered those, whose pathology specimens showed a high expression of REV-7 immunohistochemical staining in the nuclei of TGCT cells. Interestingly, 8 out of 16 REV-7 positive patients (50.0%) and 8 out of 48 REV-7 negative patients (16.7%) had chemoresistant disease. This difference was statistically significant (p = 0.016). It becomes, thus, clear that chemoresistant disease percentages were significantly higher in REV-7 positive patients (Fig. 3). REV-7 positive patients had significantly younger age, lower percentage of seminoma and higher percentages of yolk sac tumor, teratoma, and embryonal carcinoma characteristics in their orchiectomy specimens. Furthermore, REV-7 positive percentages were significantly higher in patients with non-seminoma tumors and in those with high-risk factors for stage I non-seminoma. Moreover, the percentage of positive REV-7 immunohistochemical expression was significantly lower in T1 patients and in M0 patients, according to TNM staging. On the contrary, the percentage of positive REV-7 expression was significantly higher in stage II or III patients, according to IGCCCG, as well as in patients with metastasis at diagnosis. Finally, the percentage of positive REV-7 expression was significantly higher in patients who received advanced-disease chemotherapy and in those with residual disease > 1cm after treatment. The proportion of patients who were not disease-free at last follow-up as well as those who had died, was significantly higher in REV-7 positive patients, as it is presented in Table 4.

**Table 3:** Association of REV-7 with participants’ demographics and clinical characteristics

	REV7		*Ρ*
	Negative	Positive	
	*n* (%)	*n* (%)	
Age (years), mean (SD)	38.7 (10.1)	28.6 (11.4)	0.001^‡^
Resistant/sensitive			
Chemoresistant disease	8 (50)	8 (50)	0.016^ + + ^
Chemosensitive disease	40 (83.3)	8 (16.7)	
Type			
Pure non-seminoma	13 (52)	12 (48)	0.002^ + ^
Pure seminoma	29 (93.5)	2 (6.5)	
Mixed non-seminomatous tumor	6 (75)	2 (25)	
Seminoma (%), median (IQR)	100 (0.5–100)	0 (0–0)	< 0.001^‡‡^
Choriocarcinoma (%), median (IQR)	0 (0–0)	0 (0–0)	0.223^‡‡^
Yolk sac tumor (%), median (IQR)	0 (0–0)	2.5 (0–20)	0.004^‡‡^
Teratoma (%), median (IQR)	0 (0–0)	7.5 (0–50)	< 0.001^‡‡^
Embryonal carcinoma (%), median (IQR)	0 (0–17.5)	45 (0–55)	0.019^‡‡^
High-risk factors			
No	13 (92.9)	1 (7.1)	< 0.001^ + ^
Yes (for stage I seminoma)	23 (92)	2 (8)	
Yes (for stage I non-seminoma)	12 (48)	13 (52)	
Side of orchiectomy			
Right	23 (74.2)	8 (25.8)	0.566^ + + ^
Left	20 (71.4)	8 (28.6)	
Both	5 (100)	0 (0)	
Tumor (T)			
1	16 (94.1)	1 (5.9)	0.040^ + + ^
2	20 (62.5)	12 (37.5)	
3	12 (80)	3 (20)	
Nodes (N)			
0	34 (85)	6 (15)	0.112^ + + ^
1–3	8 (61.5)	5 (38.5)	
Metastasis (M)			
0	29 (90.6)	3 (9.4)	0.011^ + ^
1a/1b/1c	17 (63)	10 (37)	
IGCCCG stage at diagnosis			
Ι	27 (87.1)	4 (12.9)	0.030^ + ^
ΙΙ/ΙΙΙ	21 (63.6)	12 (36.4)	
Metastasis at diagnosis			
No	26 (89.7)	3 (10.3)	0.014^ + ^
Yes	22 (62.9)	13 (37.1)	
Type of chemotherapy			
Advanced disease	20 (62.5)	12 (37.5)	0.030^ + ^
Adjuvant	26 (86.7)	4 (13.3)	
Residual disease > 1 cm			
No	39 (83)	8 (17)	0.022^ + + ^
Yes	9 (52.9)	8 (47.1)	

+ Pearson’s chi-square test, +  + Fisher’s exact test, ‡Student’s *t* test, ‡‡Mann–Whitney test

**Table 4:** Patients’ outcomes according to REV-7 immunohistochemical expression

		REV-7		Ρ +
		Negative	Positive	
		*n* (%)	*n* (%)	
Relapse				
	No	41 (85.4)	14 (87.5)	> 0.999
	Yes	7 (14.6)	2 (12.5)	
Disease-free at last follow-up				
	No	5 (10.6)	8 (50.0)	0.002
	Yes	42 (89.4)	8 (50.0)	
Death				
	No	47 (100.0)	12 (75.0)	0.003
	Yes	0 (0.0)	4 (25.0)	

+ Fisher’s exact test

**Fig. 4:**
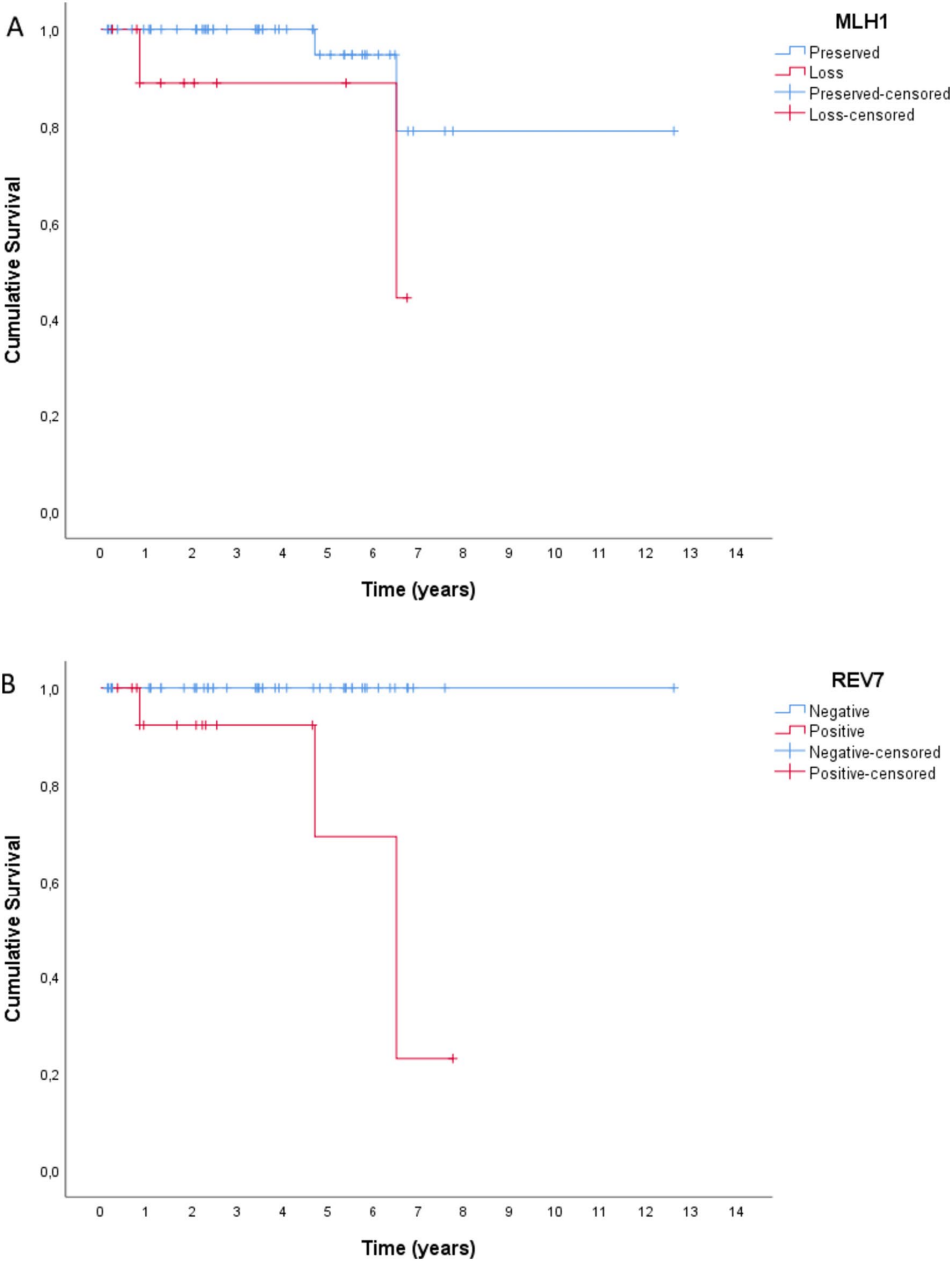
**A** Kaplan–Meier curves for survival according to MLH1 immunohistochemical expression. **B** Kaplan–Meier curves for survival according to REV-7 immunohistochemical expression

During follow-up, 4 patients (6.3%) died, while mean survival time was 10.6 years (SE = 0.9 years). Their clinical characteristics are described in table S1. Patients’ survival was not significantly associated with loss of MLH1 expression (p_log-rank_ = 0.112), as it is shown in Fig. 4A. However, it was found that patients with REV-7 positive immunohistochemical expression in their orchiectomy specimens had a significantly lower survival (p_log-rank_ = 0.001), as it is shown in Fig. 4B. Patients’ relapse was not significantly associated with loss of MLH1 expression (p_log-rank_ = 0.773), nor with REV-7 positive expression(p_log-rank_ = 0.996). Multivariate Cox regression revealed that higher percentage of teratoma in patients’ orchiectomy specimens was significantly associated with greater hazard, as it is shown in Table 5. Finally, patients with positive REV-7 expression had 14.76 times greater hazard compared to patients with negative REV-7 expression, as it is also presented in Table 5.

**Table 5:** Multiple survival analysis results, via Cox regression models

		HR (95% CI) +	*Ρ*
Teratoma (%)		1.04 (1.01–1.08)	0.021
REV-7^1^	Negative	reference	
	Positive	14.76 (1.22–178.30)	0.034

+ Hazard Ratio (95% Confidence Interval)

## Discussion

In the current study, we investigated the predictive value of immunohistochemical markers for TGCT, in a search for widely accessible methods of prediction of resistance. Other groups have also investigated the correlation between loss of MLH1 expression and chemoresistance of TGCTs. Olasz et al. investigated loss of MLH1 expression along with MLH1 gene methylation rates in 36 chemosensitive and 15 chemoresistant TGCT cases. The authors reported that although MLH1 gene methylation was frequent in TGCTs and significantly associated with loss of MLH1 expression, neither MLH1 gene methylation, MMR deficiency and MSI were associated with various clinicopathological parameters [17]. In accordance to these findings, several groups have investigated the role of MSI and MMR in TGCTs, failing to find an association with clinical outcomes [18–20]. Only Mayer et al. reported an association of MSI with chemoresistance in TGCTs, in a small however sample size (n = 11) [21]. Interestingly, when evaluating MMR deficiency, loss of MLH1 expression is often associated with the concurrent loss of PMS2 expression. This finding is expected since these are the two key proteins that form the mutLa heterodimer, which is essential for signaling downstream MMR events [8]. In this context, we also searched the literature for loss of PMS2 in TGCTs. Dum et al. studied the immunohistochemical expression of MLH1, PMS2, MSH2 and MSH6 on a tissue microarray, which contained 574 seminomas. They reported MMR deficiency, as evidenced by loss of MLH1 and PMS2 expression, in only one seminoma [22]. Finally, loss of MLH1 expression could be attributed to epigenetic events, including MLH1 promoter methylation, or germline mutations, such as those met in Lynch syndrome [23, 24].

Regarding the trends of REV-7 immunohistochemical expression in TGCTs, there is only limited evidence in the literature. Positive REV-7 immunohistochemistry has been reported in various types of cancer on human tissues, including ovarian, breast, prostate, esophageal, colorectal, lung and skin cancer and lymphomas. In most of these reports, high REV-7 immunochemical expression was associated with faster disease progression, resistance to available management options and worse prognosis in the majority of patients [25–32]. In contrast, REV7 depletion has been shown to enhance chemosensitivity in TGCT cells, due to the accumulation of DNA double-strand breaks and the activation of apoptosis [33]. These results indicate that immunohistochemical staining of REV-7 protein could potentially be used as a predictive tissue marker in certain cases [25–33].

Sakurai et al. also investigated the immunohistochemical expression of REV-7 protein in 78 testicular cancer tissue samples (53 seminomas, 11 embryonal carcinomas, three were yolk sac tumors, eight teratomas and three malignant lymphomas). In accordance to with our findings, regarding the normal adjacent tissues (non-cancer tissues), the authors reported REV-7 expression in germ cells in the seminiferous tubules, mainly in the nuclei. However, in their study 72 out of 78 tissue samples showed positive REV-7 expression. Interestingly, all the seminomas, the embryonal carcinomas and the yolk sac tumors were REV-7 positive. On the contrary, only four out of the eight included teratomas and one out of the three malignant testicular lymphomas tested positive for REV-7 expression. As already mentioned, in our study 16 (8 chemosensitive and 8 chemoresistant) TGCTs were REV-7 positive. A possible explanation for this difference in our findings is the samples’ heterogeneity. Their sample consisted mostly of seminomas (53 out of 78 specimens). In our study 31 seminomas, 25 non-seminomas and 8 mixed tumors were analyzed. Moreover, the authors did not provide any information about clinicopathological data and patients’ characteristics, because their initial aim was to investigate whether inactivation of REV-7 could increase chemosensitivity and overcome acquired chemoresistance in TGCTs. More specifically, they reported that REV-7 inactivation led to increased chemosensitivity to cisplatin and doxorubicin, by promoting DSB accumulation and apoptotic pathways, while it also recovered chemosensitivity in cisplatin-resistant cancer cells. In that way, they showed that REV-7 could potentially represent an ideal molecular target for managing chemoresistant cases of testicular germ cell tumors [33].

Another difference was that we used different antibodies. Sakurai et al. used the Anti-MAD2B (REV7) antibody (BD Biosciences, Franklin Lake, NJ, USA), while we used the Anti–REV7 antibody (Abcam, Cambridge, UK). Moreover, in their study REV-7 expression was mainly reported in the nuclei of cancer cells, as shown in the images of their article. On the contrary, we reported positive REV-7 expression in both the cytoplasm and nuclei of certain cancer cells, but mainly in the nuclei (Fig. 2). This trend was also reported for germ cells in the seminiferous tubules in our study, which could represent a positive control (Fig. 2). According to the manufacturer’s instructions (Abcam, Cambridge, UK), immunohistochemical expression can be evaluated in both the nucleus and the cytoplasm of target cells. Although, as already mentioned, there are limited reports of REV-7 immunostaining applications in human cancer tissues [25–33], immunoexpression of a protein in different cellular compartments could potentially indicate that it is engaged in different or even opposite biological functions [34]. The clinical significance of REV-7 expression in different cellular compartments needs to be elucidated, while the understanding of the biological phenomena in which this protein is engaged may further contribute towards that direction. Finally, discrepancies between our findings can be attributed to patients’ characteristics and to reviewers’ subjective interpretation.

This paper is not without limitations. To begin with, the number of included patients was relatively small (n = 64), while they were not equally allocated to the two groups (48 chemosensitive cases versus 16 chemoresistant ones). Another important limitation of our study was its retrospective design and the patients’ characteristics heterogeneity. Furthermore, data about the timing of orchiectomy were not available for all patients. It is thus possible, although extremely unlikely, that some patients had undergone chemotherapy treatment before orchiectomy. In these cases, positive REV-7 immunohistochemical staining could be potentially attributed to chemotherapy-induced DNA damage. Finally, immunohistochemical staining for REV-7 is not a standardized procedure in our Laboratory, while the anti-REV7 antibody that we used (Abcam, Cambridge, UK) is only used for research purposes, according to its manufacturer’s leaflet.

## Conclusion

Loss of MLH1 expression was only found to be significantly associated with lower patients’ age, while its was not associated with other clinicopathological factors and patients’ prognosis and overall survival. Moreover, it was not significantly correlated to chemoresistance. On the contrary, REV-7 positive percentages were significantly higher in patients with chemoresistant disease. Positive immunohistochemical REV-7 expression was significantly associated with various clinicopathological parameters, while it was also associated with significantly lower survival and a 14.76 times greater hazard. Our findings imply that immunohistochemical staining for REV-7 could potentially be used as a predictive tissue marker for TGCT tumors. Moreover, targeting of REV-7 protein, could represent a potential therapeutic strategy for chemoresistant TGCT cases. The implementation of well-designed studies on a larger scale is of utmost importance, in order to draw safer conclusions.

### Supplementary Information

Below is the link to the electronic supplementary material.

Supplementary file1 (DOCX 16 KB)
