# Supplementary material for: Predictive tissue markers in testicular germ cell tumors: Immunohistochemical expression of MLH1 and REV-7 proteins
Source: Int Urol Nephrol. 2024 Jan 27;56(6):1887–98. doi: 10.1007/s11255-023-03933-2 (PMC11090952; doi:10.1007/s11255-023-03933-2)
Supplement: Supplementary file 1 — Supplementary file1 (DOCX 16 KB) [file 11255_2023_3933_MOESM1_ESM.docx]

**Supplementary material**

**Table S1.** Tumor type, clinical stage and sensitivity of patients who died (n=4)

| n=4 | | n | % |
| --- | --- | --- | --- |
| Resistant/ Sensitive | Chemoresistant Disease | 4 | 25.0 |
|  | Chemosensitive Disease | 0 | 0.0 |
| Type | Pure Non-Seminoma | 4 | 16.0 |
|  | Pure Seminoma | 0 | 0.0 |
|  | Mixed Non-Seminomatous Tumor | 0 | 0.0 |
| T | 1 | 0 | 0.0 |
|  | 2 | 1 | 3.1 |
|  | 3 | 3 | 20.0 |
| N | 0 | 1 | 2.5 |
|  | 1-3 | 3 | 25.0 |
| M | 0 | 0 | 0.0 |
|  | 1a/ 1b/ 1c | 4 | 15.4 |
| IGCCCG stage at diagnosis | Ι | 0 | 0.0 |
|  | ΙΙ/ ΙΙΙ | 4 | 12.5 |
| Metastasis at diagnosis | No | 0 | 0.0 |
|  | Yes | 4 | 11.8 |
